# Proteomic profiling of the monothiol glutaredoxin Grx3 reveals its global role in the regulation of iron dependent processes

**DOI:** 10.1371/journal.pgen.1008881

**Published:** 2020-06-11

**Authors:** Selma S. Alkafeef, Shelley Lane, Clinton Yu, Tingting Zhou, Norma V. Solis, Scott G. Filler, Lan Huang, Haoping Liu

**Affiliations:** 1 Department of Biological Chemistry, University of California, Irvine, California, United States of America; 2 Department of Biochemistry, Faculty of Medicine, Kuwait University, Kuwait City, Kuwait; 3 Department of Physiology & Biophysics, University of California, Irvine, California, United States of America; 4 Division of Infectious Diseases, Los Angeles Biomedical Research Institute at Harbor-UCLA Medical Center, Torrance, California, United States of America; 5 David Geffen School of Medicine at UCLA, Los Angeles, California, United States of America; Stanford University School of Medicine, UNITED STATES

## Abstract

Iron is an essential nutrient required as a cofactor for many biological processes. As a fungal commensal-pathogen of humans, *Candida albicans* encounters a range of bioavailable iron levels in the human host and maintains homeostasis with a conserved regulatory circuit. How *C*. *albicans* senses and responds to iron availability is unknown. In model yeasts, regulation of the iron homeostasis circuit requires monothiol glutaredoxins (Grxs), but their functions beyond the regulatory circuit are unclear. Here, we show Grx3 is required for virulence and growth on low iron for *C*. *albicans*. To explore the global roles of Grx3, we applied a proteomic approach and performed *in vivo* cross-linked tandem affinity purification coupled with mass spectrometry. We identified a large number of Grx3 interacting proteins that function in diverse biological processes. This included Fra1 and Bol2/Fra2, which function with Grxs in intracellular iron trafficking in other organisms. Grx3 interacts with and regulates the activity of Sfu1 and Hap43, components of the *C*. *albicans* iron regulatory circuit. Unlike the regulatory circuit, which determines expression or repression of target genes in response to iron availability, Grx3 amplifies levels of gene expression or repression. Consistent with the proteomic data, the *grx3* mutant is sensitive to heat shock, oxidative, nitrosative, and genotoxic stresses, and shows growth dependence on histidine, leucine, and tryptophan. We suggest Grx3 is a conserved global regulator of iron-dependent processes occurring within the cell.

## Author summary

Mammalian pathogens occupy a diverse set of niches within the host organism. These niches vary in iron and oxygen availability. As a commensal and pathogen of humans, its ability to regulate iron uptake and utilization in response to bioavailable iron level is critical for its survival in different host environments encompassing a broad range of iron levels. This study aims to understand how *C*. *albicans* senses and responds to iron level to regulate multiple aspects of its biology. The cytosolic monothiol glutaredoxin Grx3 is a critical regulator of *C*. *albicans* iron homeostasis and virulence. Taking a proteomic approach, we identified a large list of Grx3 associated proteins of diverse functions, including iron-sulfur trafficking, iron homeostasis, metabolism redox homeostasis, protein translation, DNA maintenance and repair. In support of these protein associations, Grx3 is important for all these processes. Thus, Grx3 is a global regulator of iron homeostasis and other iron dependent cellular processes.

## Introduction

Iron is an essential nutrient for almost all living organisms with critical roles in many biological processes including respiration, metabolism, and DNA synthesis and repair. While sufficient intracellular iron level is essential for life, excess intracellular iron can also be toxic as it can lead to the generation of reactive oxygen species (ROS) [1, 2]. For pathogens, bioavailable iron level is dictated by the host environment. *Candida albicans*, a commensal and pathogenic fungus of humans, is characterized by its ability to inhabit a variety of distinct host niches that encompasses a wide range of environmental iron bioavailability, including the bloodstream where most host iron is sequestered in the form of hemoglobin and transferrin, and the mammalian gastrointestinal tract, where iron levels are high as a result of dietary consumption [2]. Therefore, the ability to regulate iron acquisition and utilization in response to environmental iron levels is critical for *C*. *albicans* pathogenesis and survival.

To that end, *C*. *albicans* utilizes a conserved transcriptional regulatory circuit to maintain iron homeostasis. In iron-replete conditions, the conserved GATA family transcription factor Sfu1 directly represses iron uptake genes and the Cys_6_Zn_2_ transcription factor Sef1 [3–5]. Conversely, in low iron conditions, Sef1 activates the expression of iron uptake genes and the highly conserved CCAAT binding protein (CBP) transcription factor Hap43, which represses iron utilization genes and *SFU1* [4, 6–8]. The roles of Sfu1 and Hap43 on the regulation of iron homeostasis are highly conserved across a broad range of fungi including *S*. *pombe* [9, 10], *H*. *capsulatum* [11, 12], and *C*. *neoformans* [13, 14]. However, how *C*. *albicans* senses iron availability to signal the iron regulatory circuit is unknown.

Monothiol glutaredoxins (Grxs) have been implicated in the regulation of iron homeostasis circuits in model yeasts. Grxs along with the functionally similar thioredoxins (Trxs) comprise a large family of thiol-disulfide oxidoreductases that regulate the redox state of cellular proteins from bacteria to mammals [15]. Grxs can be categorized as dithiol (Class I) or monothiol (Class II) [15]. Both mono- and dithiol Grxs utilize glutathione (GSH) and NADPH in redox reactions. Monothiol Grxs display little oxidoreductase activity *in vitro* [15, 16]. Instead, they play primary roles in the biogenesis and trafficking of Fe-S clusters [15, 17]. Mitochondrial monothiol Grxs are essential for early Fe-S cluster biogenesis that is necessary for the generation of all cellular Fe-S clusters [18]. Conversely, cytosolic monothiol Grxs are required for the trafficking and insertion of mature [2Fe-2S] clusters to client apoproteins [15, 17]. Loss of these proteins results in broad dysfunction in iron metabolism and increased cytosolic iron accumulation [17, 19]. In the model yeasts *S*. *cerevisiae* and *S*. *pombe* the cytosolic monothiol paralogs Grx3/4 along with their associated functional partner, the BolA-like protein Bol2/Fra2, are crucial for the iron-responsive regulation of their respective iron regulatory circuits [15, 19–24]. Grx3/4 functions in Fe-S trafficking by forming a heterodimer with the cytosolic BolA protein Bol2/Fra2, and they are thought to transfer the bound [2Fe-2S] cluster to downstream target proteins [15, 17, 19, 22]. Most studies have focused on Grxs regulation of the iron homeostasis circuit in model yeasts [24–28]. Whether these roles are conserved in *C*. *albicans* and what other proteins cytosolic monothiol Grxs regulate are yet to be determined.

Using a proteomic approach supported with mutational analysis, we identify a large number of Grx3 targets that function in diverse biological processes, most of which interact with Grx3 independently of iron status. Further analysis of Grx3 targets revealed Grx3 regulates the iron homeostasis circuit on multiple levels and is essential for full amplification of iron-regulated gene expression in response to iron level. This is the first such genome-wide proteomic approach to identify Grx3 targets. The broad range of Grx3 targets signifies the importance of Grx3 in a diverse array of biological processes that require Fe-S clusters to function.

## Results

### *C*. *albicans grx3* mutant is sensitive to iron depletion

**Fig 1:**
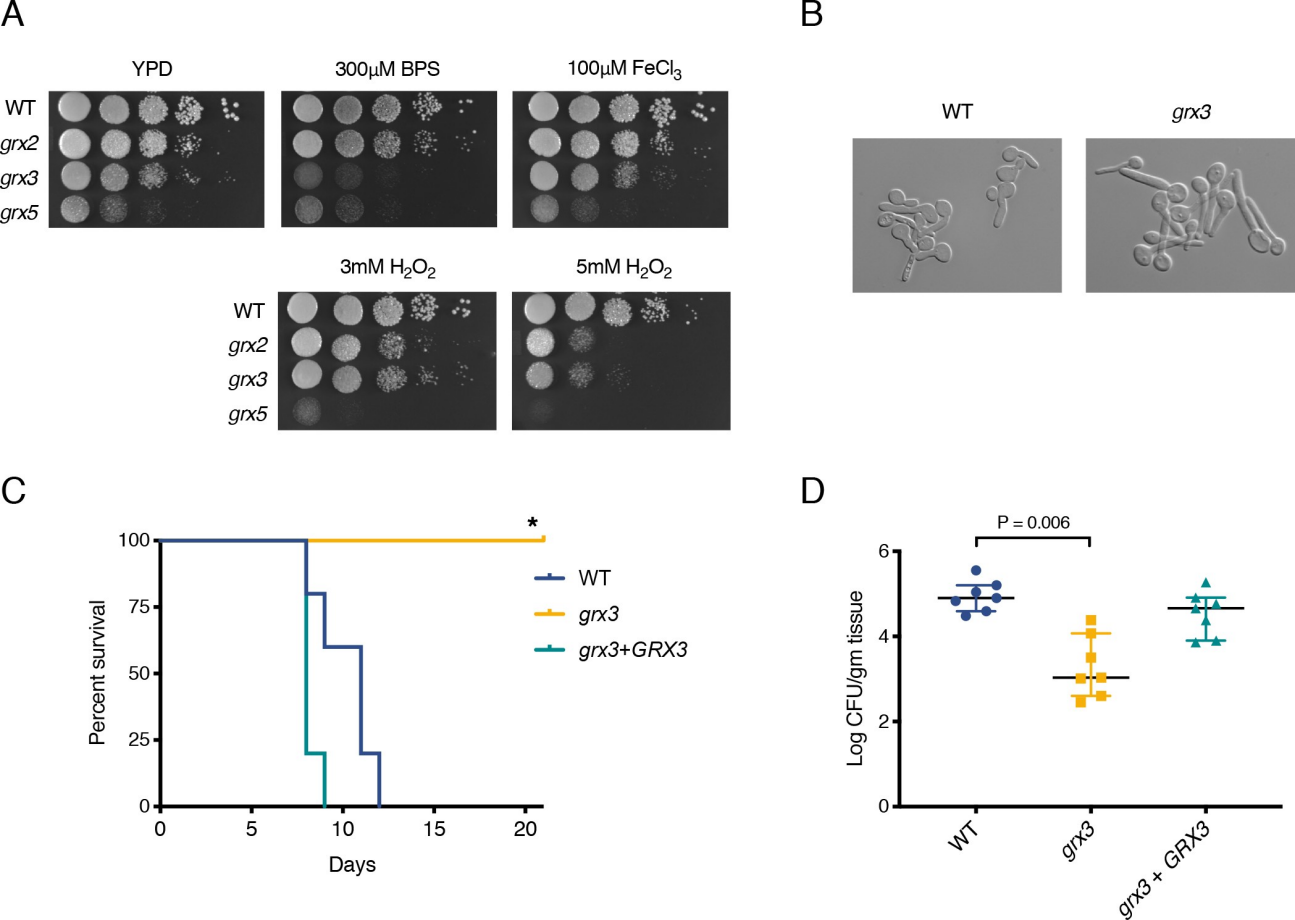
Grx3 is required for low iron stress and *C*. *albicans* pathogenicity in murine models of infection. **A.** Growth of glutaredoxin mutants under oxidative and iron stresses. Wild-type (WT) (HLY4494), *grx2* (HLY4491), *grx3* (HLY4492), and *grx5* (HLY4493) were spotted with 10-fold serial dilutions onto YPD plates containing 300μM BPS, 100μM FeCl_3_, 3mM H_2_O_2_, or 5mM H_2_O_2_ and grown for 2 days at 30°C. **B.** Hyphal initiation of WT and *grx3*. Saturated overnight cultures of WT (HLY4494) and *grx3* (HLY4492) cells were diluted 1:100 into fresh, pre-warmed YPD and grown for 1hr at 37°C and imaged. **C.** Survival of mice infected intravenously with the indicated strains. 5 mice per strain were injected in the lateral tail vein with WT (HLY4568), *grx3* (HLY4565), or *grx3* cells complemented with *pTDH3-GRX3* (HLY4566). *p = 0.0021 compared to WT by the log-rank test. **D.** Oral fungal burden of mice infected with indicated strains. 7 Mice per strain were infected orally with WT (HLY4568), *grx3* (HLY4565), or *grx3* + *pTDH3-GRX3* (HLY4566) cells. The median values and interquartile ranges are plotted as horizontal bars. Statistical significance was determined by the Wilcoxon rank sum test.

To identify the functional homolog of Grx3/4 in *C*. *albicans*, we compared the effects of oxidative and iron stresses on the growth of three *grx* mutants, the dithiol glutaredoxin mutant *grx2* and the monothiol glutaredoxin mutants *grx3* and *grx5*. We spotted cells onto YPD plates containing the iron chelator bathophenanthroline disulfonic acid (BPS) to simulate a reduced-iron environment. The *grx3* mutant showed a strong sensitivity to low iron stress on BPS-containing plates (Fig 1A, top panel). In contrast, the *grx2* and *grx5* mutants showed no additional growth defect on BPS-containing plates in comparison to YPD. None of the mutants showed sensitivity to high iron stress in the form of excess FeCl_3_ The *grx2* and *grx5* mutant showed sensitivity to both 3mM and 5mM H_2_O_2_, while the *grx3* mutant showed sensitivity to only 5mM H_2_O_2_ (Fig 1A, bottom panel). This is consistent with published data that Grx2 and Grx3 play roles in the oxidative stress response [29–31]. The *grx5* mutant had a growth defect even on YPD without H_2_O_2_, consistent with the predicted mitochondrial localization and functions of Grx5. Therefore, of the *C*. *albicans* glutaredoxin mutants tested, only the *grx3* mutant was sensitive to low iron level (Fig 1A).

### Grx3 is required for *C*. *albicans* virulence in the murine host

Filamentation is a key contributing factor to *C*. *albicans* pathogenicity [32], therefore we tested if the *grx3* mutant was defective in hyphal formation. The *grx3* mutant behaved similarly to the wild-type strain, and was able to form robust filaments with nearly 100% of *grx3* cells developing germ tubes by one hour after dilution into fresh YPD at 37°C (Fig 1B). This result suggests that intracellular iron levels do not directly influence hyphal induction.

To test the role of Grx3 in virulence *in vivo*, we utilized two murine models of candidiasis. In a disseminated model of candidiasis, mice infected with wild-type cells did not survive past day 12. In contrast, all mice infected with the *grx3* mutant strain were still alive by day 21 (Fig 1C). The rescued *grx3* mutant strain carrying a single copy of *GRX3* driven by the *TDH3* promoter was able to fully complement the *grx3* mutant phenotype, and mice infected with the rescued strain all died by 9 (Fig 1C, S1 Fig). Therefore, the *grx3* mutant shows attenuated virulence in a model of disseminated candidiasis. We also tested the pathogenicity of the *grx3* mutant in the mouse model of oropharyngeal candidiasis (OPC). Mice infected with the *grx3* mutant showed decreased oral fungal burden compared to those infected with the wild-type and *GRX3* rescued strains (Fig 1D). Therefore, Grx3 is important for *C*. *albicans* pathogenicity in the murine host during both hematogenously disseminated and mucosal infection. How Grx3 contributes to virulence is unknown. To address this, we took a global proteomic approach to shed light on Grx3 functions.

### Proteomic profiling of Grx3 interacting proteins identifies Grx interacting proteins in Fe-S assembly and delivery, the iron homeostasis regulon, and other processes

Grx3 is suggested to deliver Fe-S clusters to recipient proteins. Fe-S cluster trafficking and handoff are likely dynamic processes, consequently interactions between Grx3 and target proteins may be difficult to capture. Therefore we utilized *in vivo* cross-linked His-Biotin-His (HBH) tandem affinity purification, coupled with label-free quantitative mass spectrometry to identify Grx3 interacting proteins in living cells [33–36]. A functional *pMAL2*-driven Grx3-HBH fusion construct in the *grx3* mutant strain (S2 Fig) was used for purifications. Any interacting proteins that appeared in the untagged control samples were excluded from analysis. To further reduce the incidence of false positives arising non-specifically from HBH purification, we also excluded any proteins that appeared in our previously published mass spectrometry datasets derived from HBH-purifications of the unrelated transcriptional regulators opaque-specific Wor1 and general co-repressor Tup1 [33]. We further reduced background noise by eliminating orthologs of *S*. *cerevisiae* proteins present in the CRAPome, a database of non-specific affinity purification contaminant proteins [37]. We identified 343 proteins present in at least 2 or more out of 4 Grx3-HBH purifications, 64 of which were identified in all 4 samples (S1 Table).

**Fig 2:**
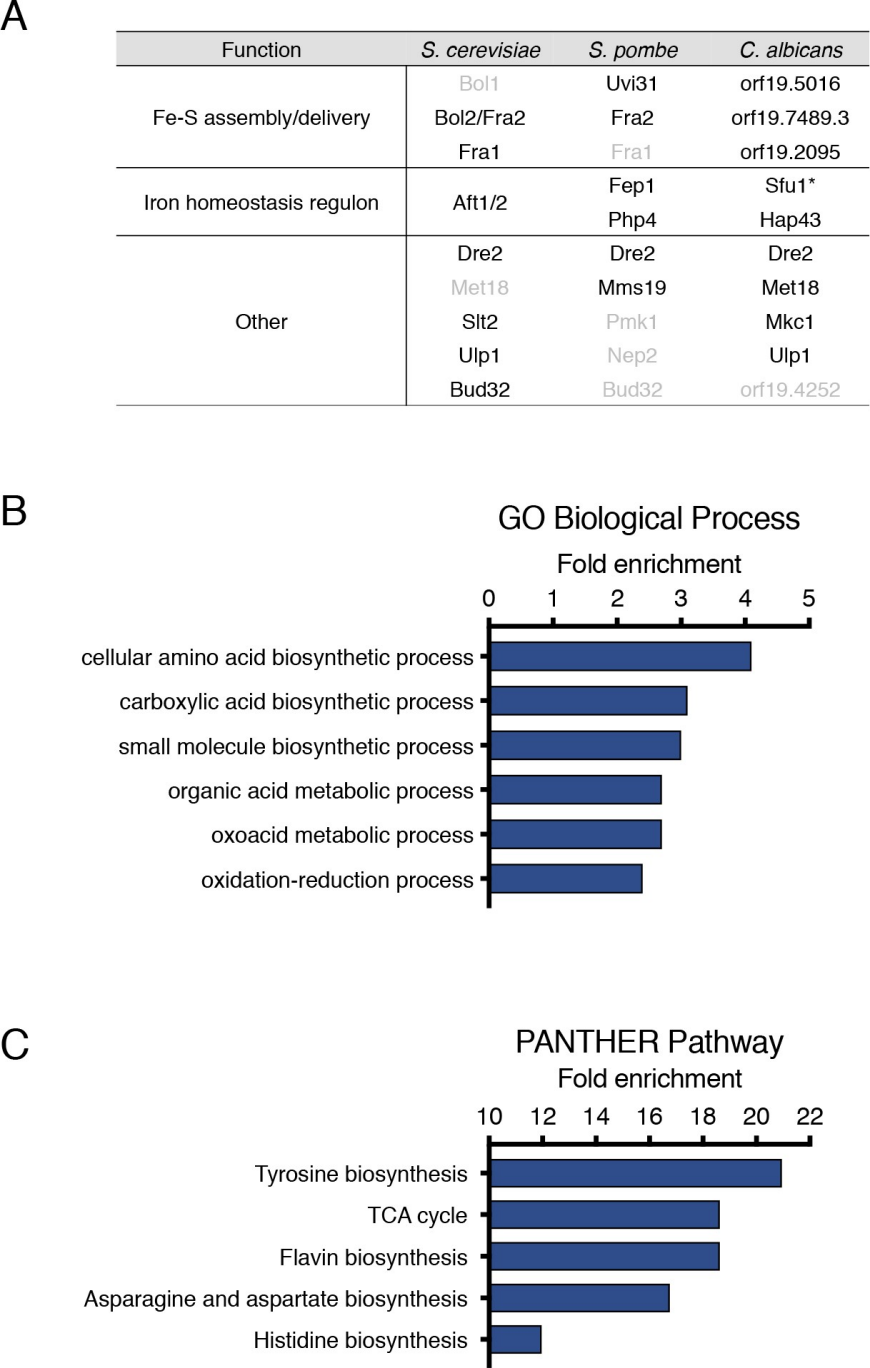
Grx3 interacting proteins function in a wide array of biological processes. **A.** Table of known glutaredoxin-associated proteins in *S*. *cerevisiae* and *S*. *pombe*. These proteins are also observed in *C*. *albicans* Grx3-HBH mass spectrometry experiments. Proteins are arranged by homology and/or shared function. Verified physical interactions are indicated in black text while grey text indicates no known interaction. Proteins marked with * were also observed in Tup1-HBH purifications. Physical interactions observed in *S*. *cerevisiae* and *S*. *pombe* were identified using the Saccharomyces Genome Database, BioGrid, and PomBase [38–40]. **B.** Gene Ontology analysis by biological process of Grx3 interacting proteins identified by mass spectrometry. Proteins observed in 2 or more out of 4 total samples were analyzed using the Candida Genome Database [41]. Categories with a fold enrichment < 2 and a FDR P value of < 0.05 were excluded. Full GO results are presented in S2 Table. **C.** Biological pathways found enriched PANTHER Pathways analysis. Categories with a fold enrichment of <10 and an FDR P value of <0.05 were excluded.

In order to assess the validity and sensitivity of our proteomic approach, we compiled a list of verified physical interactors of Grx3 homologs in *S*. *cerevisiae* and *S*. *pombe* (Fig 2A) based on the Saccharomyces Genome Database, Pombase, and BioGrid, [38–40]. We included proteins with two or more independent reports of interactions in *S*. *cerevisiae*, and only 10 proteins met the criteria from 27 high throughput physical interaction studies (BioGrid). Of the 10 validated proteins shown to physically interact with Grx3 homologs, we identified 9 *C*. *albicans* homologs in our Grx3 interaction data (Fig 2A, S1 Table). Therefore our proteomic approach is highly sensitive in capturing most of the known Grx3/4 interacting proteins. The observations of bona-fide Grx interacting proteins in our mass spectrometry data validate our proteomic method.

The most characterized of Grx3/4-interacting proteins are the Fe-S cluster binding BolA-like proteins Bol1 and Bol2/Fra2, the aminopeptidase P-like protein Fra1, and the iron homeostasis regulon proteins Aft1/2 (*S*. *cerevisiae*), Fep1, and Php4 (*S*. *pombe*) (Fig 2A) [15, 19, 24, 25, 27]. BolA-like proteins are integral to Fe-S cluster biogenesis and trafficking; Bol2/Fra2, and to a lesser extent Fra1, play crucial roles in Grx-mediated regulation of iron homeostasis circuits [15, 24]. Our observation that Grx3 interacts with the *C*. *albicans* homologs of these well-characterized BolA proteins suggests that *C*. *albicans* Grx3 likely retains similar functions as an iron trafficker as those described. Our proteomic approach also identified Hap43 and Sfu1, transcription factors in the *C*. *albican*s iron homeostasis circuit (Fig 2A) [3, 4, 7]. The identification of these Grx3 targets from our proteomic approach support the conserved role of Grx3 in the regulation of Fe-S cluster biogenesis, trafficking, and iron homeostasis *in C*. *albicans* [19, 22, 42, 43].

We performed Gene Ontology (GO) analysis to identify enriched functional categories of Grx3-associated proteins [44–46]. We found enrichment of proteins involved in a range of cellular processes including oxidation-reduction, metabolism, and amino acid biosynthetic processes (Fig 2B, S2A Table). Pathway analysis showed enrichment in flavin, histidine, tyrosine, and asparagine and aspartate biosynthetic processes (Fig 2C, S2B Table). Taken together, GO analysis of our Grx3-associated proteins is consistent with the known role of Grx3 in maintaining cellular redox homeostasis (Fig 1A) [31] and suggests a broader role for Grx3 in the regulation of multiple biological processes that are dependent on Fe-S clusters. To the best of our knowledge, this is the first such proteomic approach undertaken to identify a comprehensive listing of Grx-associated proteins *in vivo*.

### Grx3 regulates the *C*. *albicans* iron homeostasis circuit and amplifies iron-responsiveness of gene expression

**Fig 3:**
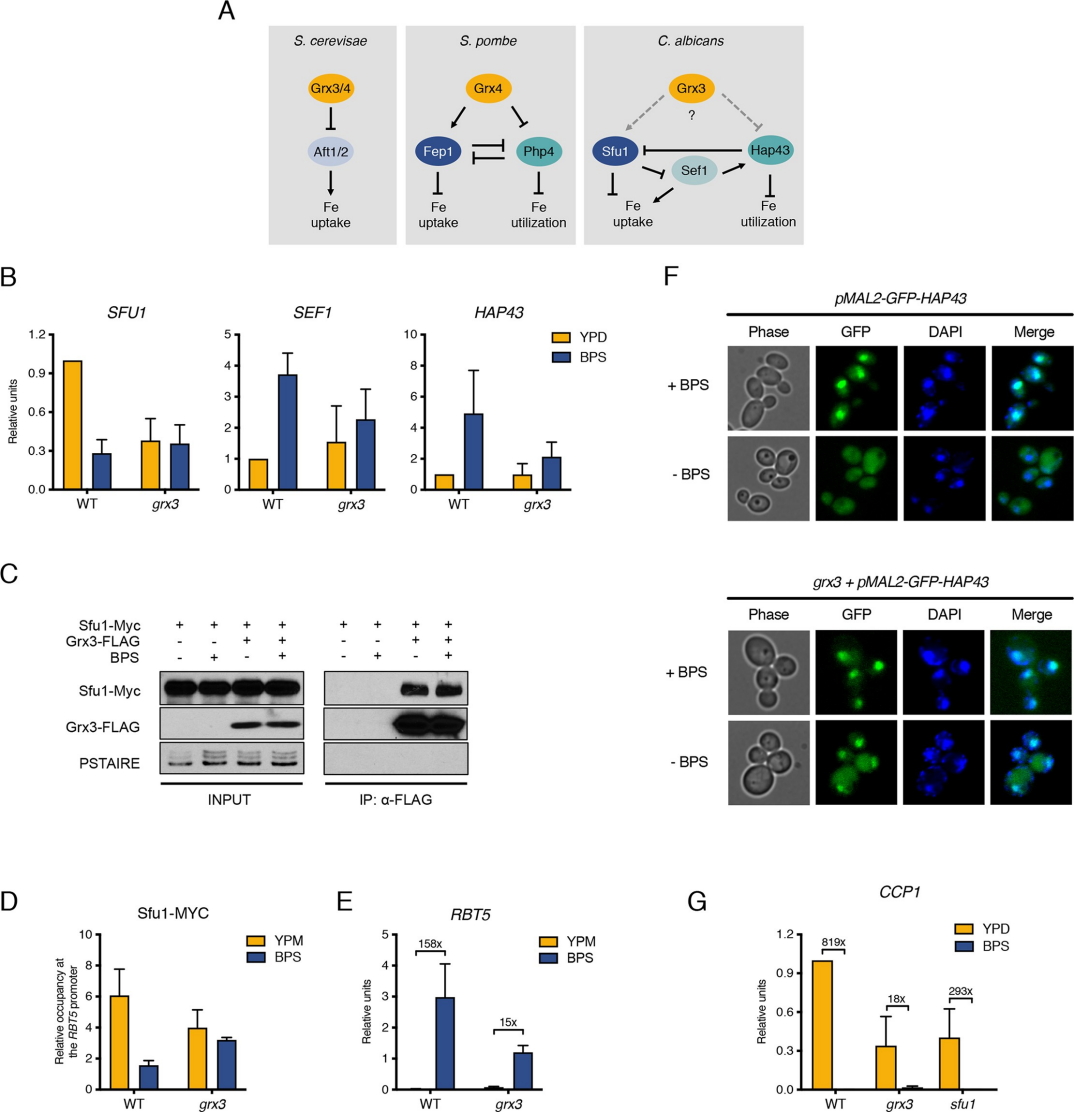
Grx3 regulates the iron homeostasis circuit in *C*. *albicans*. **A.** Current models of iron homeostasis regulation in *S*. *cerevisiae*, *S*. *pombe*, and *C*. *albicans*. **B.** Expression of *SFU1*, *SEF1* and *HAP43* in WT (HLY4494) and *grx3* mutant (HLY4492). Cells were grown in YPD media in the presence or absence of 500μM BPS. Expression level of the indicated genes was measured by qPCR and normalized to *ACT1*. Presented is the average expression level from three independent experiments with error bars representing the s.d. The same applies to **E**, **G**. **C.** Immunopreicipitation of Grx3-FLAG. Strains HLY4500 (*pGRX3-GRX3-FLAG*, *pMAL2-SFU1-MYC*) and HLY4498 (*pMAL2-SFU1-MYC*) were grown in the presence or absence of 500μM BPS at 30°C for 6hr. **D.** Chromatin immunoprecipitation of Sfu1-Myc. WT (HLY4498) and *grx3* (HLY4507) strains carrying *pMAL2-SFU1-MYC* were cultured in the presence or absence of 500μM BPS at 30°C. qPCR analysis of Sfu1 enrichment at the *RBT5* promoter was calculated by the percent input method and is presented as a ratio of the *RBT5* promoter relative to an *ADE2* control region, with error bars representing SEM. **E.** *RBT5* gene expression in WT (HLY4494) and *grx3* (HLY4492) strains. **F.** Localization of *MAL2*-driven GFP-Hap43 in *hap43* (HLY4569) and *grx3* (HLY45701). Cells were grown overnight in SCM in the presence or absence of 300μM BPS. **G.** *CCP1* expression in WT (HLY4494), *grx3* (HLY4492), and *sfu1*.

*C*. *albicans* maintains iron homeostasis by utilizing a transcriptional regulatory circuit comprised of the transcription factors Sfu1, Sef1, and Hap43 (Fig 3A) [4]. The mechanisms by which the *C*. *albicans* iron regulatory circuit is able to sense iron level are unknown, although in model yeasts monothiol glutaredoxins have been shown to functionally regulate iron homeostasis regulons in response to iron level (Fig 3A) [15, 19, 25, 28]. In *S*. *cerevisiae*, Grx3 homologs Grx3/4 regulate the localization and activity of the transcriptional activators Aft1/2 [20, 21, 23]. In *S*. *pombe*, the Grx3 homolog Grx4 directly interacts with Fep1, an Sfu1 homolog, in both iron replete and iron limited conditions to regulate Fep1 occupancy at target promoters [24, 27, 28]. Further, Grx4 regulates the activity of Hap43 homolog Php4 through a mechanism of nucleo-cytoplasmic shuttling in response to iron level [25, 26, 47]. Sfu1 and Hap43 both co-purified with Grx3 in our mass spectrometry data suggesting that Grx3 may similarly regulate Sfu1 and Hap43 activity. In wild-type cells, *SFU1* is expressed in YPD but down regulated during iron limitation in BPS; conversely, expression of *SEF1* and *HAP43* is high during iron limitation and low in YPD (Fig 3B) [4]. In the *grx3* mutant, expression levels of *SFU1*, *SEF1*, and *HAP43* were relatively unchanged between YPD and BPS treatment. This suggests that the iron homeostasis regulatory circuit is no longer responsive to change in iron level in the absence of Grx3 (Fig 3B). Our data for the first time links Grx3 with iron-responsive transcriptional regulators in *C*. *albicans*.

### Grx3 interacts with Sfu1 and is necessary for Sfu1 to associate with target promoters

To determine if Grx3 binds Sfu1 in *C*. *albicans*, we constructed a strain carrying Grx3-FLAG and *MAL2*-driven Sfu1-Myc. Immunoprecipitation of Grx3-FLAG confirmed that Grx3 and Sfu1 interact *in vivo*, both in YPM and BPS-containing media (Fig 3C). Therefore, Grx3 and Sfu1 interact independently of iron level. As Sfu1 binds target promoters to repress gene expression in iron replete conditions [4], we performed chromatin immunoprecipitation (ChIP) of maltose-induced Sfu1-Myc in wild-type and *grx3* mutant cells and assessed Sfu1 binding at the promoter of *RBT5*, a GPI-linked cell wall protein involved in hemoglobin utilization [48]. Sfu1 occupancy at the *RBT5* promoter was dependent on iron level (Fig 3D), consistent with previous observations [4]. In the *grx3* mutant, the levels of promoter bound Sfu1 were similar in high and low iron conditions: lower than wild-type in iron replete conditions but higher than wild-type in iron limiting conditions (Fig 3D). This result indicates that Grx3 regulates iron-responsive binding of Sfu1 to the *RBT5* promoter. *RBT5* expression levels correlated with Sfu1 binding (Fig 3E). *RBT5* transcription was highly induced in BPS in the wild-type cells, but the fold induction in response to iron depletion was much lower in the *grx3* mutant (Fig 3E). Thus, loss of Grx3 corresponded to decreased amplitude of iron-responsiveness of gene expression. However, *RBT5* still exhibited differential gene expression between high and low iron conditions (Fig 3E), suggesting the presence of Grx3-independent factors involved in the regulation of *RBT5* expression.

### Grx3 represses Hap43 activity by promoting cytoplasmic localization

We next assessed the role of Grx3 on Hap43 activity. Work in *S*. *pombe* has shown that the Grx3 homolog, Grx4, regulates the activity of Php4, a Hap43 homolog, by modulating Php4 localization in response to iron level [25, 26, 47]. To test if this regulation was conserved in *C*. *albicans*, we constructed a GFP-Hap43 fusion protein under the control of the *MAL2* promoter and transformed it into the *hap43* and *grx3* mutant strains. The construct was able to complement the *hap43* mutant phenotype on low concentrations of BPS (S3 Fig). When cultured in BPS-containing medium (low iron), GFP-Hap43 localized primarily to the nucleus (Fig 3G). Conversely, under iron replete conditions (SCM), GFP-Hap43 showed an increased cytosolic presence and was no longer enriched in the nucleus (Fig 3G). This iron-responsive change in GFP-Hap43 nuclear and cytoplasmic localization is at least partially dependent on Grx3, as *grx3* cells displayed nuclear localization of GFP-Hap43 in both high and low iron conditions (Fig 3G). Therefore, we suggest that Grx3 likely represses Hap43 activity by promoting Hap43 localization to the cytosol under iron replete conditions.

Expression of the Hap43 target gene *CCP1*, an iron utilization gene, was measured under iron replete and iron limited conditions (Fig 3F). Of the three iron circuit regulators, only Hap43 binds to the *CCP1* promoter, where it functions to repress *CCP1* expression in low iron conditions, making *CCP1* an ideal gene to assay Hap43 activity [4]. In wild-type cells, *CCP1* was expressed in YPD but repressed by 819-fold in BPS-containing medium (Fig 3F). *CCP1* expression was lower in the *grx3* mutant in YPD compared to the wild-type strain (Fig 3F), suggesting increased Hap43 activity as a transcriptional repressor in the absence of Grx3. Furthermore, the fold repression of *CCP1* in response to low iron in the *grx3* mutant was only 18 fold, which is 45-fold lower than in wild-type cells. In contrast to deleting *GRX3*, deleting *SFU1* only slightly reduced the fold repression of *CCP1*. Our data suggests that Grx3 represses Hap43 activity and plays a critical role in *CCP1* repression in response to iron level. This is consistent with work in that has shown Php4 activity and localization are directly regulated by Grx4 in response to iron level [25, 26, 47]. These findings support our previous observations that Grx3 controls iron homeostasis by regulating the amplitude of iron-responsive gene expression (Fig 3B and 3E).

### Grx3 contributes to the histidine, tryptophan, and leucine biosynthesis pathways

**Fig 4:**
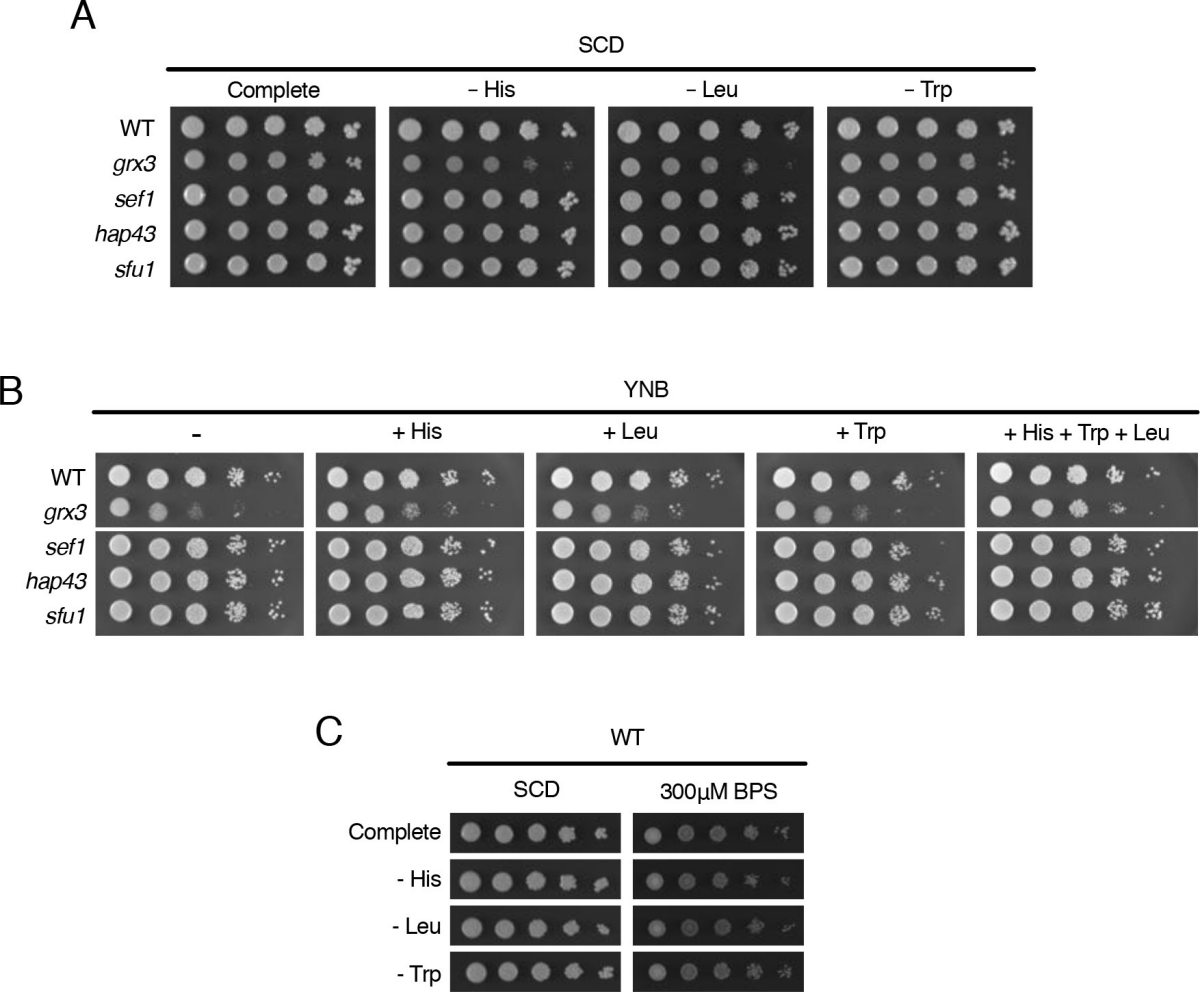
Grx3 regulates histidine, leucine, and tryptophan biosynthesis pathways. **A.** Growth of WT (HLY4568), *grx3* (HLY4492), *sef1*, *hap43*, and *sfu1* on SCD plates lacking the indicated amino acids at 30°C for 2 days. **B.** Growth of the same strains on YNB dextrose plates containing the indicated amino acids at 30°C for 2 days. **C.** Growth of the WT (HLY4568) strain on SCD or SCD with BPS plates lacking the indicated amino acids at 30°C for 2 days.

GO analysis of the Grx3 mass spectrometry data showed enrichment for general amino acid biosynthetic processes (Fig 2B) with specific enrichment for tyrosine, asparagine, aspartate, and histidine biosynthesis pathways (Fig 2C). Therefore we systematically tested which amino acids the *grx3* mutant might require for optimal growth. The *grx3* mutant displayed a growth defect on SCD plates lacking histidine, leucine, and a slight growth sensitivity to tryptophan deprivation (Fig 4A). We did not observe a growth defect on SCD media lacking any other amino acids. The *grx3* mutant grew slower than WT on minimal media (YNB), and YNB supplemented with histidine, tryptophan, and leucine was sufficient to restore *grx3* growth (Fig 4B). This function of Grx3 was independent of the iron homeostasis transcriptional circuit, as the iron circuit mutants *sfu1*, *sef1*, and *hap43* were not sensitive to histidine, leucine, or tryptophan deprivation (Fig 4A and 4B). Further, the wild-type strain was not defective on BPS-treated SCD plates lacking histidine, tryptophan, or leucine, suggesting the growth dependency of the *grx3* mutant on these amino acids is due to the role of Grx3 in regulating the activities of these pathways (Fig 4C).

This is the first report that Grx3 plays important roles in histidine, leucine, and tryptophan biosynthesis. Interestingly, in *S*. *cerevisiae*, the leucine biosynthesis pathway is regulated in response to iron level through both transcriptional and post-transcriptional regulation of the Fe-S cluster-containing enzyme Leu1, suggesting that it controls the key regulated step of leucine biosynthesis [49]. In *C*. *albicans*, *LEU1* expression is upregulated 8-fold under iron-replete conditions [4]. Our findings describe a transcription-independent role for Grx3 in regulating iron-dependent metabolic and biosynthetic processes and support a novel function of Grx3 in amino acid biosynthesis pathways as identified by our proteomic approach.

### Grx3 contributes to cellular stress responses

**Fig 5:**
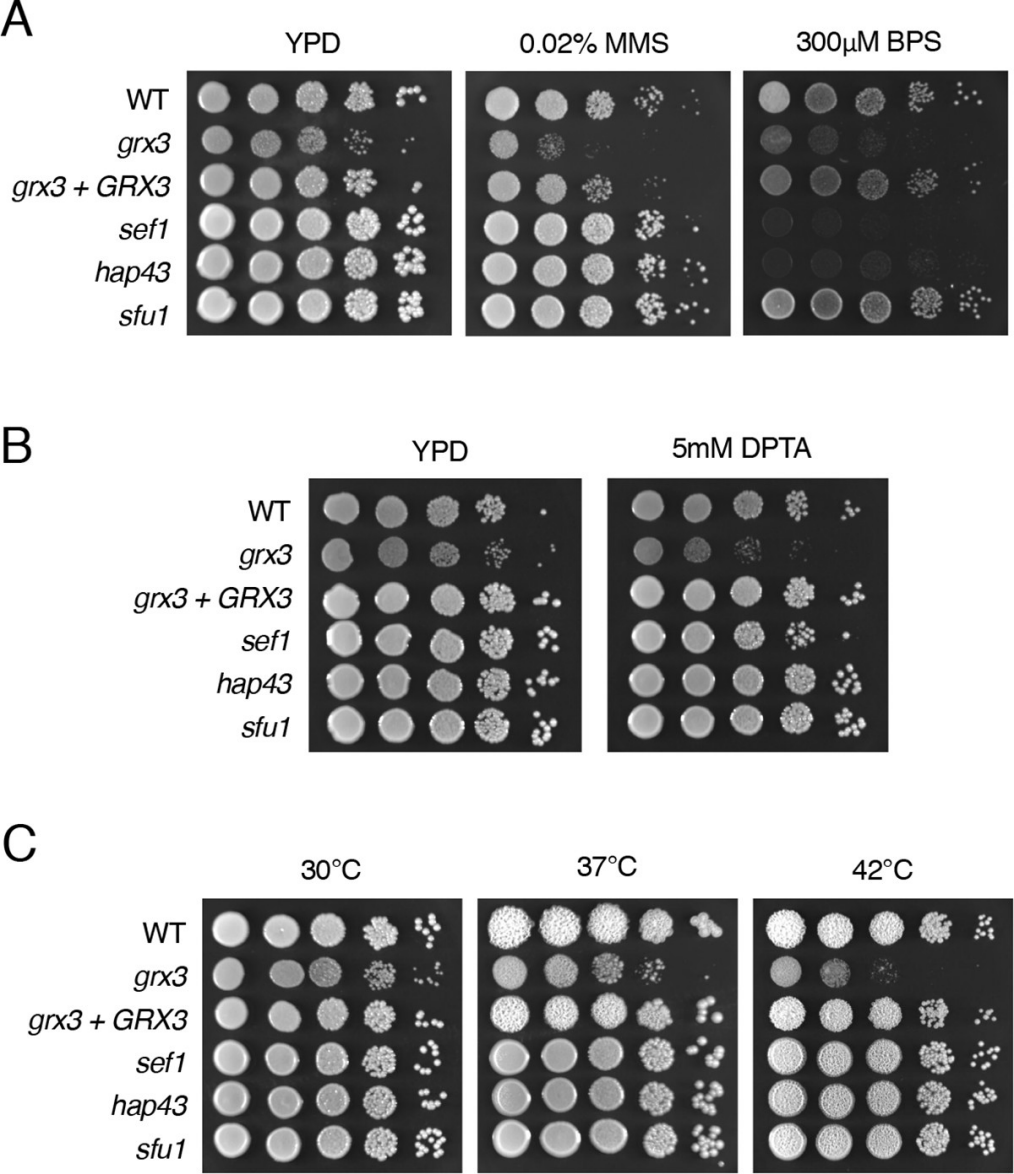
Grx3 contributes to cellular stress response. Sensitivity of *grx3* to genotoxic stress (**A),** nitrosative stress (**B**), and elevated temperature (**C**). WT (HLY4494), *grx3* (HLY4492), *grx3 + GRX3* (HLY4566), *sef1*, *hap43*, and *sfu1* were serially diluted and spotted onto YPD plates containing indicated compounds and grown at 30°C or indicated temperature for 2 days.

We next sought to experimentally assess and validate the roles of Grx3 in different processes enriched by GO analysis of our mass spectrometry data (Fig 2C, S2 Table). Fe-S cluster biogenesis has previously been implicated in DNA replication and repair pathways [50]. Defects in mitochondrial or cytosolic Fe-S cluster biogenesis result in genomic instability [51, 52]. Our mass spectrometry dataset included proteins involved in both Fe-S cluster biogenesis and DNA damage response such as Dre2 [53], Met18 (*S*. *cerevisiae* Mms19) [52, 54], Rad6 [55], and Rad51 [56]. To assess the role of Grx3 in genomic maintenance and the DNA damage response, we spotted cells onto YPD plates containing the genotoxic stress methyl methanesulfonate (MMS) and assayed for sensitivity (Fig 5A). The *grx3* mutant was sensitive to MMS, but *sef1*, *hap43*, and *sfu1* showed no growth defect in response to MMS treatment, indicating that the observed sensitivity of the *grx3* mutant to genotoxic stress is not due to dysfunction of the iron regulatory circuit (Fig 5A). As a control, the *grx3* mutant and iron homeostasis circuit mutants *sef1* and *hap43* were all unable to grow on BPS-treated plates. Therefore the role of Grx3 in maintaining genomic integrity is independent of its function in the regulation of the iron homeostasis circuit and likely occurs through its role in Fe-S cluster trafficking.

Having previously found the *grx3* mutant sensitive to oxidative stress (Fig 1A) and having observed GO enrichment in oxidation-reduction processes (Fig 2B), we tested what other types of cellular stresses Grx3 may play a role in responding to. We found the *grx3* mutant, but not the iron regulatory circuit mutants *sef1*, *hap43*, and *sfu1*, was sensitive to nitrosative stress (Fig 5B) and growth at 42°C (Fig 5C). This suggests that Grx3 contributes to the regulation of multiple stress response pathways in *C*. *albicans*. Beyond oxidation-reduction processes, we did not observe enrichment for GO categories relating to other general cellular stress response or heat shock processes (Fig 2). This is perhaps due to our stringent removal of common affinity purification contaminants from our Grx3 interaction dataset, which typically include HSP chaperones. Therefore our proteomic approach unveiled new functions for Grx3 in the regulation of cellular stress response processes independent of its role in the iron homeostasis transcriptional circuit.

## Discussion

**Fig 6:**
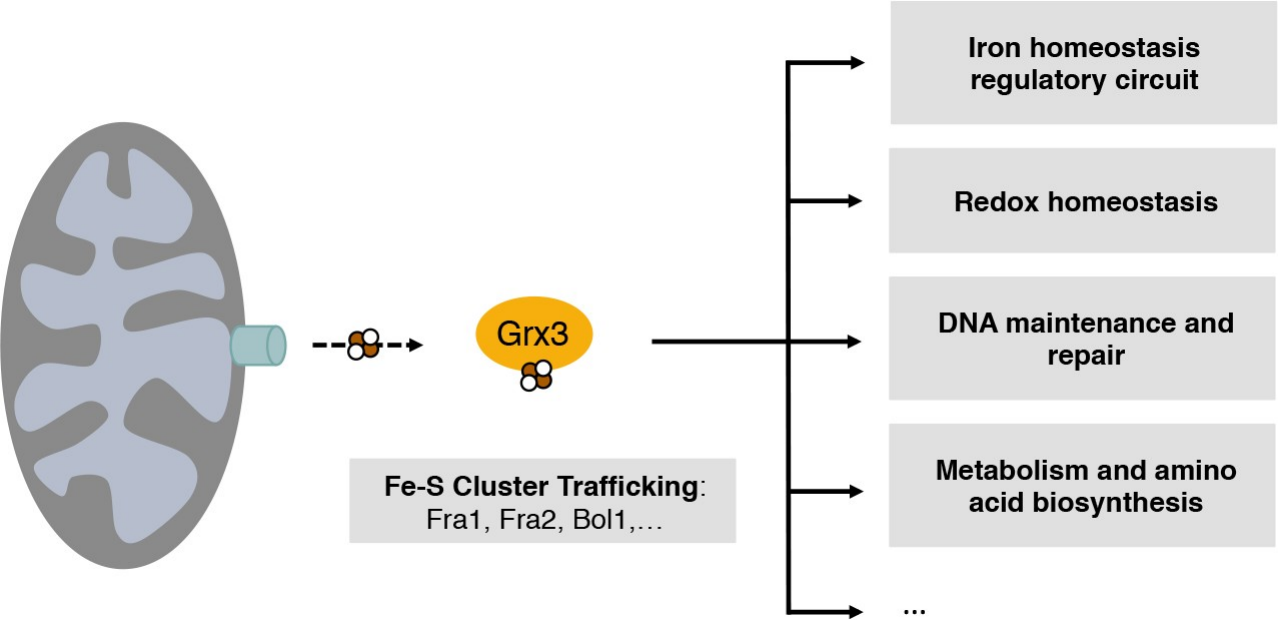
Grx3 is a global iron sensor and Fe-S carrier critical for the function of multiple Fe-S protein-dependent pathways. Cartoon of global Grx3 function. The Fe-S cluster-carrying protein Grx3 is an intracellular iron sensor and Fe-S trafficker that contributes to the function of a multitude of proteins involved in a wide range of biological processes as listed.

Here we show that the *C*. *albicans* cytosolic monothiol glutaredoxin Grx3 is essential for the regulation of not only the iron homeostasis circuit, but an array of Fe-S cluster-dependent processes occurring within the cell (Fig 6). *In vivo* cross-linked purification of Grx3 coupled with mass spectrometry enriched for interacting proteins involved in a variety of important processes including Fe-S cluster biogenesis and trafficking, iron homeostasis, redox homeostasis, metabolism, amino acid biosynthesis, and DNA maintenance and repair. The dependence of these processes on iron or Fe-S cofactors, the known function of Grxs in Fe-S cluster biogenesis and trafficking, and the fact that Grx3/4 deficiency in *S*. *cerevisiae* functionally impairs all iron-requiring processes [17] lead us to conclude that *C*. *albicans* Grx3 is a global regulator of iron responses that controls a multitude of Fe-S cluster-dependent pathways and processes (Fig 6).

One critical gap in our knowledge of how Grxs function within the cell is the lack of a global and comprehensive understanding of Grx targets. Most work, performed in model yeasts, focused either on how monothiol Grxs regulate iron homeostasis transcription factors in response to iron level or how Grxs physically interact with their BolA-like protein complex partners in Fe-S cluster trafficking. Beyond a small pool of identified Fe-S acceptors, very few Grx downstream targets are known. Our proteomic approach provides an unbiased global look at the full scope of Grx3 downstream targets and pathways Grx3 may regulate. The incorporation of *in vivo* cross-linking was key to capturing weak or dynamic interactions and provided us an extensive view of the Grx3 interactome. One drawback to this method is the high level of false positives that may arise due to non-specific affinity purification as well as increasingly sensitive instrumentation. To overcome these limitations we utilized multiple rounds of elimination using publicly available affinity purification contaminant repositories (the CRAPome) and our own previous unrelated affinity purifications [33, 37]. While stringent and possibly removing bona fide Grx3 interacting proteins (false negatives), we believe this approach lends confidence that the majority of our identified Grx3 interacting proteins represent legitimate interactions.

We identified Sfu1 and Hap43 as Grx3 interacting proteins and show that Grx3 is required for the iron-responsive regulation of the *C*. *albicans* iron homeostasis circuit, as loss of *grx3* results in the insensitivity of transcriptional outputs to iron level. Grx3 interacts with Sfu1 and controls Sfu1 occupancy at its target promoters, consistent with the finding in *S*. *pombe* that Grx4 controls promoter association of the Sfu1 homolog Fep1 [27]. In addition, Grx3 regulates Hap43 activity and localization in response to iron level. This is similar to *S*. *pombe* where Grx4 is responsible for the nucleo-cytoplasmic shuttling of the Hap43 homolog Php4 in response to iron level [25, 26, 47]. We observed Grx3 interacts with Hap43 in both iron replete and limited conditions (S1 Table), consistent with work in *S*. *pombe* that has shown Grx4 interacts constitutively with Php4 through its TRX domain but in an Fe-S cluster-dependent manner through interactions with its GRX domain [25, 26, 47]. Our work suggests this Grx3-mediated regulation of Hap43 activity is likely conserved in *C*. *albicans*. Interestingly, in the *grx3* mutant, the fold induction of downstream genes *RBT5* and *CCP1* in response to iron level was greatly reduced compared to wild-type (Fig 3E and 3F). While the transcriptional circuit of Sfu1, Sef1, and Hap43 toggles between the high and low iron response by controlling gene expression, Grx3 contributes to their regulation and amplifies the iron response. Therefore, Grx3 regulation of multiple transcriptional regulators of the iron homeostasis circuit increases the sensitivity of gene expression to iron level.

Although the iron homeostasis circuit has evolved to use different transcription factors in fungi, their regulation by Grx3 seems to be conserved. In *S*. *cerevisiae*, under iron replete conditions the Fe-S cluster-bridged Grx3/4-Bol2/Fra2 complex promotes the DNA dissociation and nuclear export of the transcription factors Aft1/2, thereby blocking Aft1/2-mediated induction of iron uptake genes [20, 21, 23, 57]. *C*. *albicans* Grx3 was recently implicated in regulating the nuclear localization of an Aft2-like protein [31], although Aft2 is not part of the iron regulatory circuit [5]. *C*. *albicans* has evolved to use Sef1 in addition to Sfu1 and Hap43 to control iron-responsive transcription [4]. *Candida glabrata* displays a hybrid system incorporating both Aft1 and Sef1 factors in the regulation of iron homeostasis [58]. The *C*. *glabrata* Grx3 homolog Grx4 likely plays a similar role in controlling iron-responsive transcription. Our study in *C*. *albicans* extends the previous findings from model organisms, linking cytosolic monothiol glutaredoxins to the regulation of their respective iron homeostasis transcriptional circuits in pathogenic fungi. Recently, a Grx3 homolog in another pathogenic fungus, *Cryptococcus neoformans*, was identified and characterized [59]. Using a transcriptomic approach, *C*. *neoformans* Grx4 was found to regulate iron homeostasis, virulence, metabolism, and cellular stress response, consistent with our proteomic findings presented here and work performed in other model organisms.

Bol2/Fra2 (orf19.7489.3) and Bol1 (orf19.5016) are both identified in our mass spectrometry data (S1 Table). Cytosolic Grx3/4-Bol2/Fra2 heterodimers have been shown to function as Fe-S cluster chaperones that facilitate Fe-S incorporation into apoproteins in the cytosol from yeast to mammalian cells [19, 24, 42]. It is likely *C*. *albicans* also uses the Grx3-Bol2/Fra2 complex as an Fe-S cluster chaperone for [2Fe-2S] trafficking to target proteins. While Bol2/Fra2 displays cytosolic localization, Bol1 and Bol3 occupy the mitochondria where they function in late stage iron-sulfur cluster (ISC) assembly and participate in the biogenesis of [4Fe-4S] clusters [60]. It is proposed that Grx3/4 functions downstream of the mitochondrial ISC and is a cytosolic recipient of [2Fe-2S] clusters generated from this pathway [15]. Although widely known for its role as a cytosolic or nuclear protein, Grx3/4 has also been shown to display mitochondrial localization [19, 60]. What role mitochondrial-associated Grx3 may play is unknown, but our data suggests it might involve the mitochondrial Bol1 in this function.

How does Grx3 sense cellular iron level and regulate the activity of so many different client proteins? It has been demonstrated in model organisms that the conserved cytosolic monothiol glutaredoxins are essential for inserting Fe-S clusters into target proteins [17, 24]. Cells without functional Grx3/4 accumulate iron but are unable to use it [17]. The Fe-S center of Grx3/4 is critical for both iron delivery and signaling [17, 24]. Substituting the active site cysteine with serine or alanine in the GRX domain of Grx3/4 blocks the ability of Grx3/4 to sense and deliver iron to its client proteins [17]. In addition, the Fe-S cluster binding sites of client proteins are also essential for their function [24]. Therefore, we speculate that glutaredoxins and BolA-like proteins govern client protein activity in response to iron level by regulating the Fe-S cluster status of the client protein. Our work suggests a broad role for Grx3 in overall iron regulation of an assortment of biological processes and pathways. Due to the ubiquitous nature of Grxs across all kingdoms of life, the functional relationships between monothiol Grxs and the biological processes described in this study should show broad conservation throughout the living world.

## Materials and methods

### Growth media and culturing conditions

Strains were grown at 30°C in either YEP (1% yeast extract, 2% peptone), synthetic complete (SC) medium (0.17% Difco yeast nitrogen base w/o ammonium sulfate, 0.5% ammonium sulfate, complete supplement amino acid mixture), or YNB medium (0.17% Difco yeast nitrogen base w/o ammonium sulfate, 0.5% ammonium sulfate) plus 2% either dextrose or maltose as indicated. Iron depletion was induced by growth in media containing 100μM, 300μM, or 500μM bathophenanthroline disulfonic acid (BPS), as indicated. High iron stress was induced through addition of 100μM FeCl_3_. All HBH purification cultures were grown in media supplemented with 4μM biotin.

### Plasmid and strain construction

All strains used in this study are presented in S3 Table. All primers used are presented in S4 Table.

#### *grx2*, *grx3*, and *grx5* strains

Strains were taken from the GRACE library and loss of the tetracycline transactivator was selected for by growth on 5-FOA plates [61].

#### pMAL2-SFU1-13xMYC

*SFU1* was amplified by primers 1 and 2. The resulting amplicon was inserted by Gibson assembly into an XbaI and PacI digested BES119 vector backbone [62] resulting in p1248. The resulting plasmid was digested with AscI for insertion into the *ADE2* locus and transformed into the CaSS1 wild-type strain resulting in HLY4498 or into the *grx3* mutant strain (HLY4492) resulting in HLY4507.

#### pGRX3-GRX3-FLAG

The *GRX3* coding sequence and promoter were amplified using primers 3 and 4. The FLAG sequence was amplified from the pMAL2-WOR1-FLAG plasmid (p1142) [33] using primers 5 and 6. The final pGRX3-GRX3-FLAG plasmid (p1252) was inserted by Gibson assembly into a PstI and KpnI digested pWOR1-GFP plasmid (p1029) carrying the NAT resistance gene. The resulting plasmid was digested with HindIII for insertion into the *pGRX3* locus of HLY4498, yielding HLY4500.

#### pMAL2-GRX3-HBH

*GRX3* was amplified using primers 7 and 8. The *GRX3* amplicon were inserted by Gibson assembly into an XbaI and MluI digested pMAL2-WOR1-HBH plasmid (p1251) [33]. The resulting plasmid was digested with AscI for insertion into the *ADE2* locus and transformed into the *grx3* mutant (HLY4492), generating the strain HLY4559.

#### pMAL2-GFP-HAP43

*HAP43* was amplified with primers 15 and 16 and inserted by Gibson assembly into an MluI and KpnI digested pMAL2-GFP-CPH2 plasmid (p1028) [63]. The resulting plasmid was digested with AscI for integration into the *ADE2* locus and transformed into *hap43* and *grx3*, yielding HLY4569 and HLY4570, respectively.

#### WT, Ura+ His+ strain

An empty BES116 plasmid [62] was digested with AscI and integrated into the *ADE2* locus of the wild-type strain (HLY4494). Subsequently, the *HIS3* gene along with flanking upstream and downstream regions was amplified using primers 9 and 10 and transformed into the His+ wild-type strain resulting in HLY4568.

#### *grx3* Ura+ strain

An empty BES116 vector was linearized with AscI and integrated into the *ADE2* locus of the *grx3* strain (HLY4492), yielding HLY4565.

#### pTDH3-GRX3

933bp of the *TDH3* promoter was amplified and fused to the *GRX3* coding sequence using primers 11–14. The resulting PCR fusion was inserted by Gibson assembly into a NotI and KpnI digested pMAL2-WOR1-HBH (p1251) plasmid. The resulting plasmid was linearized with AscI and integrated into the *ADE2* locus of *grx3* (HLY4492) yielding the strain HLY4566.

### Spot testing

Overnight cultures of the indicated strains were washed three times with H_2_O and diluted to an OD_600_ of 1.0. Strains were then serially diluted 10-fold, and 2.5–5μL was spotted onto plates of the indicated media. Plates were grown for 2–3 days at 30°C. For low iron stress, strains were spotted onto YPD plates containing 300μM or 500μM bathophenanthroline disulfonic acid (BPS) as indicated. For high iron stress, strains were spotted onto YPD plates with 100μM FeCl_3_. For oxidative stress, cells were treated to 3mM or 5mM H_2_O_2_. Genotoxic stress was induced through treatment with 0.02% methyl methanesulfonate (MMS), and nitrosative stress induced with 5mM dipropylenetriamine (DPTA) NONOate. SC spot plates were supplemented with all amino acids unless indicated otherwise. YNB spot plates were supplemented with arginine due to auxotrophic requirements of the *sef1*, *hap43*, and *sfu1* mutants [64].

### Quantitative PCR

RNA samples were prepared using the Quick-RNA Miniprep Kit from Zymo Research. cDNA was synthesized using the iScript Reverse Transcription Kit (Bio-Rad) and qPCR was performed using iQ SYBR Green Supermix (Bio-Rad). Primers used for qPCR are listed in S3 Table.

### Immunoprecipitation

Cultures were grown overnight at 30°C in YPM then diluted into fresh YPM in the presence or absence of 500μM BPS. Cultures were grown for another 6hr then harvested, washed twice with ice cold H_2_O, and lysed. Lysates were clarified, input sample was saved, and the lysates were incubated at 4°C with 50μL anti-FLAG M2 affinity gel (Sigma) for 2hr, washed five times with lysis buffer, and eluted in TE buffer with 1% SDS. Samples were resolved by SDS-PAGE and analyzed by western blot.

### Western blotting

Samples were resolved on 10% SDS-PAGE gels, transferred onto nitrocellulose membranes, blocked, and probed for the proteins of interest. FLAG-tagged proteins were detected with mouse monoclonal anti-FLAG M2 (Sigma), MYC-tagged proteins were detected with HRP-conjugated anti-MYC antibody (Roche), and PSTAIRE (Cdc28) was detected using a rabbit polyclonal antibody (Santa Cruz). All non-HRP conjugated antibodies were detected by either goat anti-mouse or goat anti-rabbit HRP-conjugated secondary antibodies (Bio-Rad).

### Chromatin Immunoprecipitation

ChIP was performed as described, with slight modifications [65]. Strains were cultured overnight in YPM at 30°C then diluted into fresh YPM with or without 500μM BPS and grown for 6hr. Samples were then fixed with 1% formaldehyde for 15 min, quenched with 125mM glycine for 5 min, and washed twice with ice cold PBS. DNA was sheared by sonication for 10 cycles of 20s at 40s intervals using a Bioruptor (Diagenode) and 1% input samples were saved. Sfu1-FLAG was immunoprecipitated using anti-FLAG M2 affinity gel (Sigma). DNA from input and IP samples was quantified by qPCR. Enrichment of tagged samples was quantified by the percent input method comparing the region of interest over the control region *ADE2* and further normalizing to an untagged strain, with values representing at least three independent experiments and error bars representing the SEM. Primers used are listed in S3 Table.

### Microscopy

For *pMAL2-GFP-HAP43* visualization, cells were grown in SCM with or without 300μM BPS and imaged. Cells were stained with DAPI (4’, 6-diamidino-2-phenylindole) for nuclear localization. Images were taken using an inverted Zeiss Axio Observer.Z1 microscope (Carl Zeiss MicroImaging) equipped with an X-Cite series 120 mercury lamp. DAPI, GFP fluorescence, and cell morphology were imaged using the DAPI, GFP, and DIC channels, respectively.

### HBH purification for mass spectrometry

Protein purification was performed as described by Alkafeef et al., with modifications [33]. Cultures of a *grx3* strain carrying pMAL2-GRX3-HBH and an untagged control strain were grown in YPM overnight then diluted into fresh YPM with or without 500μM BPS, grown for 6hr or 16hr as indicated, cross-linked in 1% formaldehyde for 10 min, quenched with 125mM glycine for 5 min, then centrifuged, washed twice with H_2_O, and either flash frozen and stored at -80°C or lysed with Buffer A (8M urea, 300mM NaCl, 0.5% NP-40, 50mM sodium phosphate pH 8, 50mM Tris pH 8, 20mM imidazole, 1mM PMSF, 1 Roche Complete EDTA-free protease inhibitor tablet). All other steps were performed as previously described.

### Mass spectrometry and data analysis

LC-MS/MS analysis was carried out using an UltiMate 3000 UHPLC (Thermo Fisher Scientific) coupled on-line to an Orbitrap Fusion Lumos Tribrid mass spectrometer (Thermo Fisher Scientific). Each duty cycle comprises an FT scan mass spectrum (m/z 375–1500, resolution of 120,000 at *m/z* 400) followed by data-dependent MS/MS spectra at top speed for 3 s. Raw data for both analyses were searched against a database consisting of *Candida* proteins (PA.orf_trans_all_assembly_21_2009_0306_v2 with a total of 6243 protein entries) using MaxQUANT. The mass tolerance for parent ions and fragment ions were set as ± 20 ppm and 0.5 Da, respectively. Trypsin was set as the enzyme, and a maximum of two missed cleavages were allowed. Protein N-terminal acetylation and methionine oxidation were selected as variable modifications. Protein and PSM FDR was set as 0.01. LFQ protein quantitation was calculated using a minimum ratio count of 2 peptides [66, 67]. All raw data was deposited and can be accessed at ftp://MSV000084168@massive.ucsd.edu with Firefox (username, if prompted: MSV000084168; password: GRX3BPS2019).

### Gene Ontology analysis

Proteins observed in 2 or more out of 4 purifications were subject to Gene Ontology analysis using both the Candida Genome Database (CGD) GO Slim tool and PANTHER Pathway [41, 46, 68]. Results with a FDR P value < 0.05 were excluded. A full table of all enriched GO terms is presented in S2 Table.

### Hyphal initiation

Cells were grown overnight at 30°C in YPD media then diluted 1:100 into fresh pre-warmed YPD and grown at 37°C for 1hr. Cells morphology was then assessed by microscopy.

### Virulence testing

The virulence of the *grx3* mutant in mouse models of hematogenously disseminated candidiasis and OPC was determined as previously described (PMID: 21085601; 25693184;11600377; 22402633). Briefly, hematogenously disseminated candidiasis was induced by inoculating 5 male Balb/c mice per strain with 5 x 10^5^ organisms via the lateral tail vein. The mice were monitored 3 times daily for survival and moribund mice were humanely euthanized. For the OPC model, each *C*. *albicans* strain was tested in 7 mice. Each mouse was immunosuppressed with cortisone acetate, anesthetized, and then inoculated by placing a swab saturated with 10^6^ *C*. *albicans* cells per ml under the tongue for 75 min. After 5 days of infection, the mice were sacrificed and their tongues were harvested, weighed, homogenized, and quantitatively cultured.

## Supporting information

S1 Table Grx3 interacting proteins identified by mass spectrometry. Wild-type (HLY4494) and *grx3* cells carrying *pMAL2-GRX3-HBH* (HLY4559) were grown in YPM in the presence or absence of 500μM BPS before formaldehyde cross-linking and purification under denaturing conditions. Proteins observed in untagged control samples, previously performed unrelated HBH purifications, and an online contaminant repository called the CRAPome were all excluded from the dataset [33, 37]. Grx3-HBH interacting proteins identified by mass spectrometry are ranked in decreasing order first by count of samples each protein was observed in then by decreasing average iBAQ (intensity-based absolute quantification) value as calculated by MaxQUANT [67, 69]. Protein function was taken from the Candida Genome Database [41].
(XLSX)

S2 Table GO analysis of Grx3 interacting proteins. A complete listing of enriched GO categories of Grx3-HBH co-purified proteins presented in S1 Table. Proteins observed in 2 out of 4 or more samples were analyzed using the CGD GO Finder Slim tool by biological process (**A**) or PANTHER Pathways (**B**). Results with a FDR P value < 0.05 were excluded.
(XLSX)

S3 Table Strains used in this study. (XLSX)

S4 Table Primers used in this study. (XLSX)

S1 Fig *pTHD3-GRX3* rescues the *grx3* phenotype. WT (HLY4568), *grx3* (HLY4565), and *grx3* transformed with *pTDH3-GRX3* (HLY4566) were 10-fold serially diluted and spotted onto YPD plates containing 300μM or 500μM BPS and grown at 30°C for 2 days.
(TIFF)

S2 Fig The Grx3-HBH tag is functional. WT (HLY4494), *grx3* (HLY4492), and *grx3* transformed with *pMAL2-GRX3-HBH* (HLY4559) were serially diluted and spotted onto YPM plates with 300μM or 500μM BPS and grown at 30°C for 2 days.
(TIFF)

S3 Fig The GFP-Hap43 fusion protein is functional at low concentrations of BPS. WT (HLY4494), *hap43*, and *hap43* transformed with *pMAL2-GFP-HAP43* (HLY4569) were 10-fold serially diluted and spotted onto YPM plates in the presence or absence of 100μM BPS and grown at 30°C for 2 days.
(TIFF)
