# Supplementary figures and images for: Proteomic profiling of the monothiol glutaredoxin Grx3 reveals its global role in the regulation of iron dependent processes
Source: PLoS Genet. 2020 Jun 11;16(6):e1008881. doi: 10.1371/journal.pgen.1008881 (PMC7319344; doi:10.1371/journal.pgen.1008881)

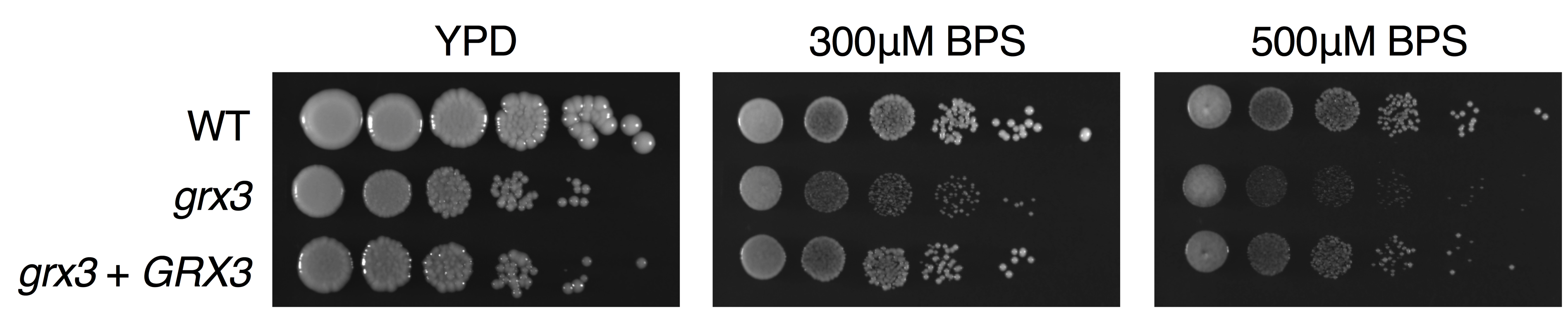

Supplement: S1 Fig — WT (HLY4568), grx3 (HLY4565), and grx3 transformed with pTDH3-GRX3 (HLY4566) were 10-fold serially diluted and spotted onto YPD plates containing 300μM or 500μM BPS and grown at 30°C for 2 days. (TIFF) [file pgen.1008881.s005.tiff]

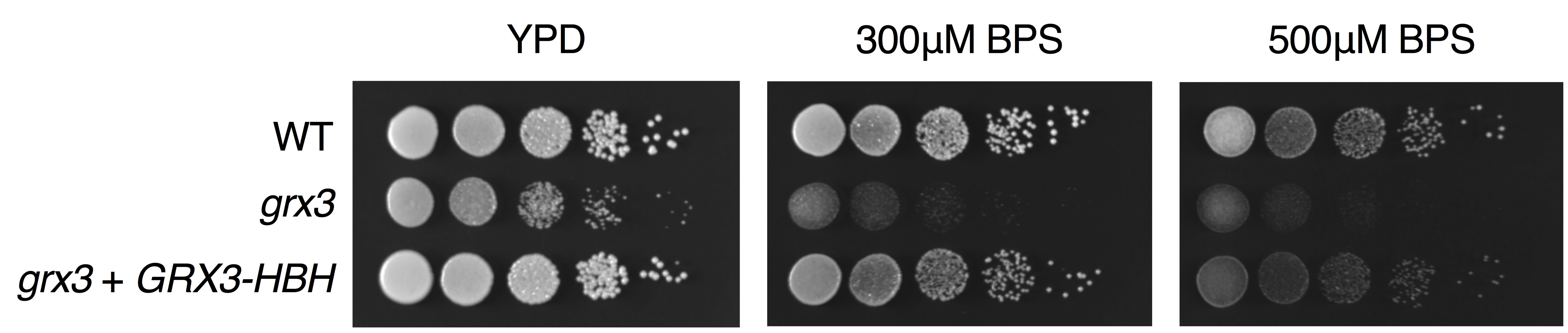

Supplement: S2 Fig — WT (HLY4494), grx3 (HLY4492), and grx3 transformed with pMAL2-GRX3-HBH (HLY4559) were serially diluted and spotted onto YPM plates with 300μM or 500μM BPS and grown at 30°C for 2 days. (TIFF) [file pgen.1008881.s006.tiff]

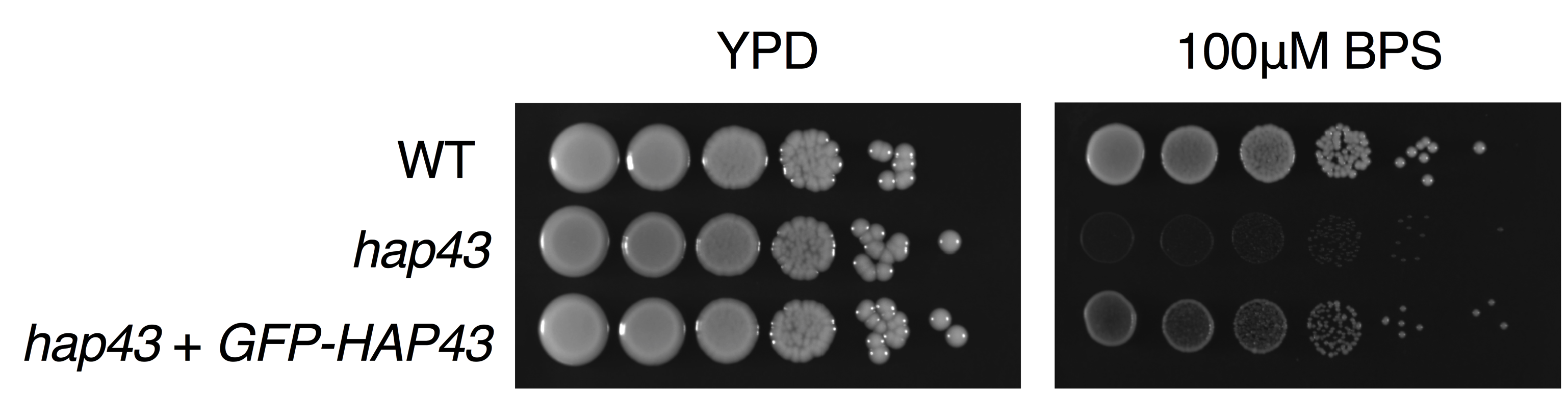

Supplement: S3 Fig — WT (HLY4494), hap43, and hap43 transformed with pMAL2-GFP-HAP43 (HLY4569) were 10-fold serially diluted and spotted onto YPM plates in the presence or absence of 100μM BPS and grown at 30°C for 2 days. (TIFF) [file pgen.1008881.s007.tiff]
